# Supplementary material for: Staufen1 links RNA stress granules and autophagy in a model of neurodegeneration
Source: Nat Commun. 2018 Sep 7;9:3648. doi: 10.1038/s41467-018-06041-3 (PMC6128856; doi:10.1038/s41467-018-06041-3)
Supplement: Supplementary file 1 — Supplementary Information [file 41467_2018_6041_MOESM1_ESM.pdf]

Staufen1 links RNA stress granules and autophagy in a model of neurodegeneration

Paul *et al.*

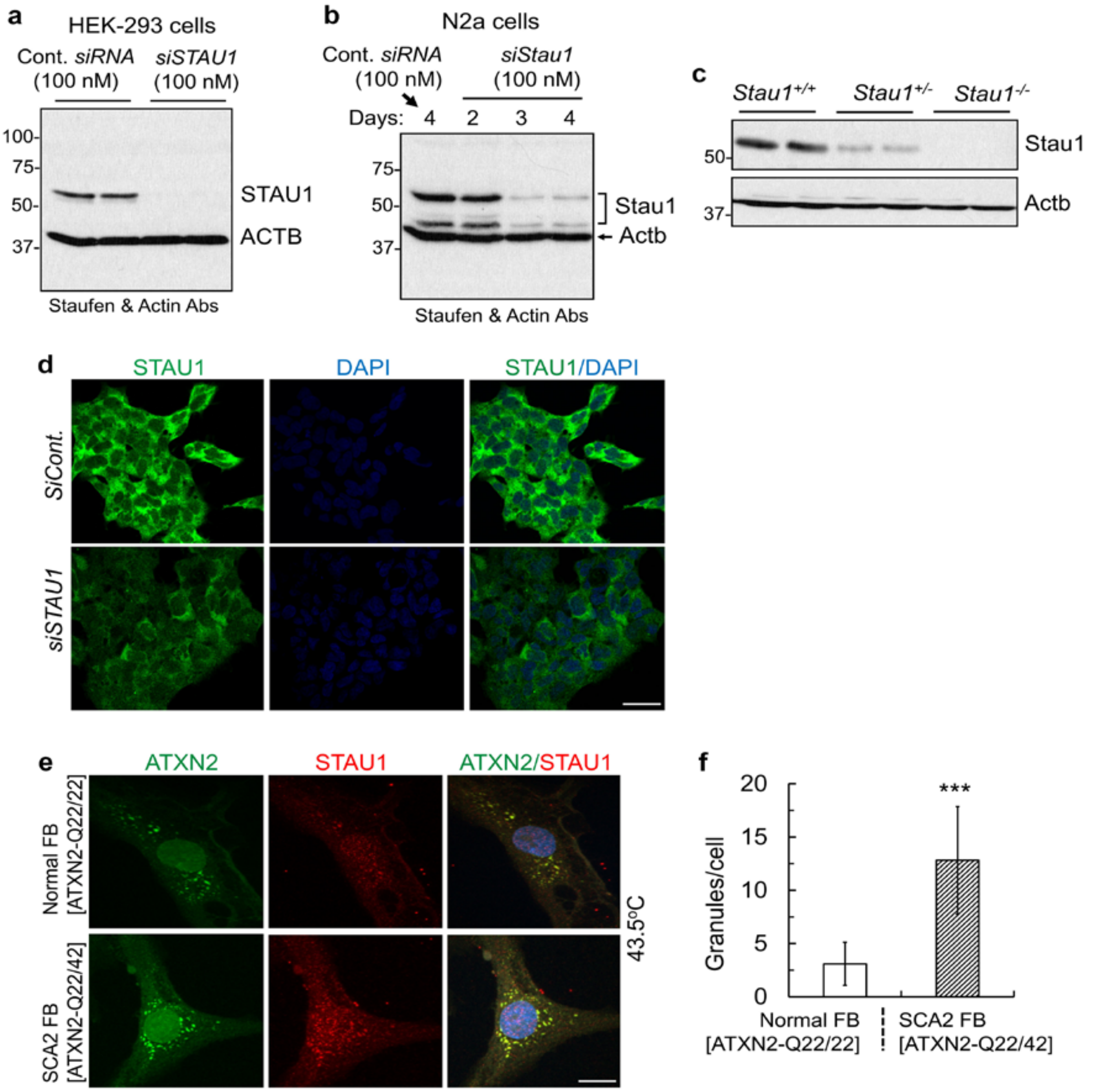

Supplementary Figure 1 | Characterization of the Staufen antibody. (**a,b**) The Staufen antibody demonstrated specificity for the detection of endogenous human and mouse Staufen1. Human HEK-293 cells or mouse N2a neuroblastoma cells were transfected with control and human or mouse Staufen1 siRNAs. For human *siSTAU1*, HEK-293 cells were harvested at 4 days post-transfection (**a**), and for mouse *siStau1*, N2a cells were harvested at the indicated time point in days (**b**). Protein extracts were analyzed by western blotting to verify Staufen1 silencing. (**c**) Western blot analyses show reduction of Stau1 in *Stau1*$^{+/-}$ and *Stau1*$^{-/-}$ mouse cerebella compared with wildtype *Stau1*$^{+/+}$ mice (two animals per group). According to the NCBI database STAU1 is translated as multiple variants. Staufen antibody detects human STAU1 variant ~63 kDa, mouse Stau1 variants (~49 and ~55 kDa) in N2a cells, and ~55 kDa for mice (**a-c**). β-Actin used as an internal loading control and representative blots are shown. (**d**) HEK-293 cells were transfected with control or *STAU1* RNAis (100 nM) and STAU1 (green) silencing was evaluated by

immunostaining with Staufen antibody 4 days post-electroporation. (**e,f**) ATXN2-STAU1 granules during stress. Immunostaining of normal and SCA2 (ATXN2-Q22/42) FBs with ATXN2 and Staufen Abs during heat shock (43.5 °C for 1 hr). Both normal and SCA2 FBs are positive for ATXN2-STAU1 granules (**e**). Numbers of ATXN2-STAU1 positive SGs in SCA2 FBs during heat shock stress are significantly greater than in heat shock stressed normal FBs (**f**). 34 normal and 30 SCA2-FBs were used for analyses. Data are mean ± SD, ***$P < 0.001$, Student t-test. Scale bar, 30 µM.

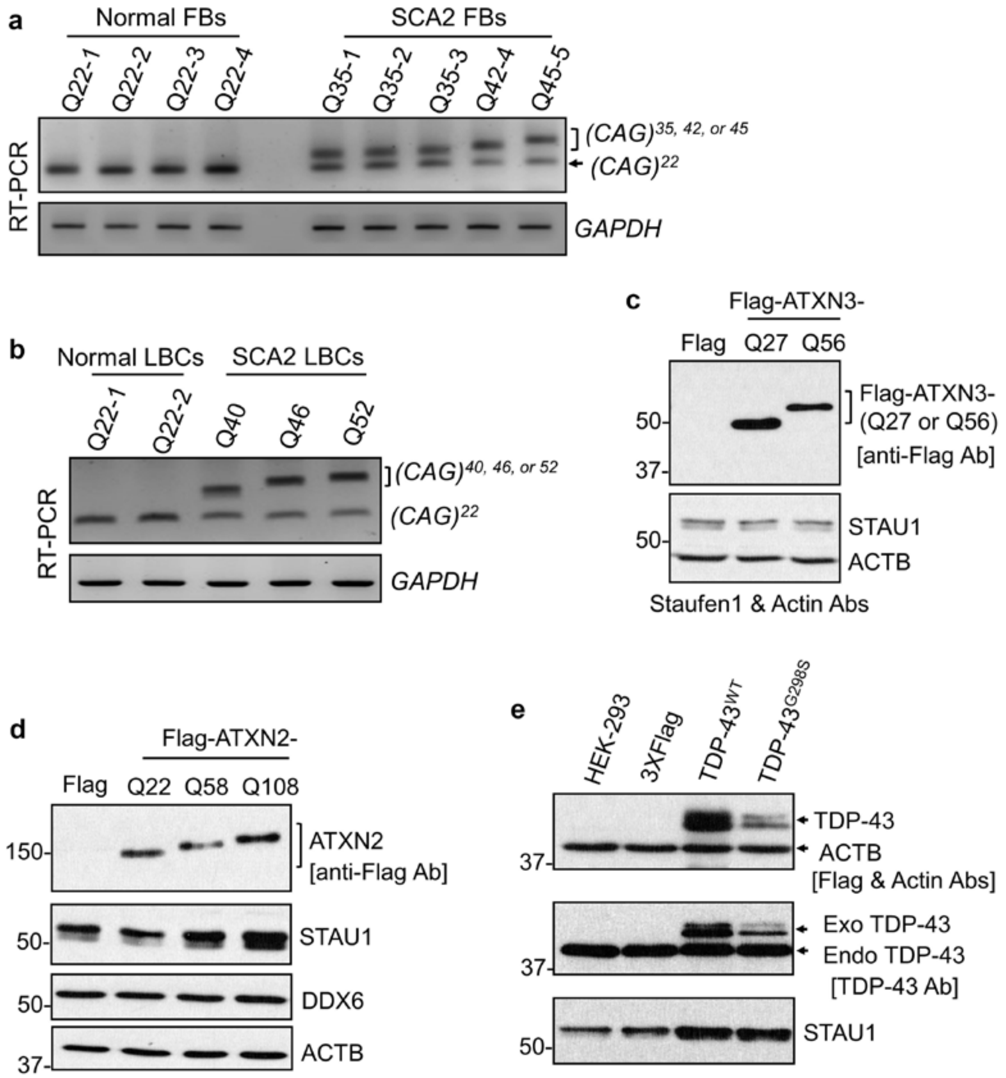

Supplementary Figure 2 | CAG repeat validations in SCA2 cells and recapitulation of STAU1 abundances by the expression of mutant ATXN2 or TDP-43. (**a,b**) Validation of CAG repeats in *ATXN2* in SCA2 cells. Total RNAs were isolated from FBs and LBCs from SCA2 patients and normal control individuals, and subjected to RT-PCR analyses using primers specifically amplifying the human *ATXN2* CAG repeat. The *GAPDH* gene was amplified as an internal control. (**c**) Overexpression of mutant ATXN3 shows unaltered STAU1 level compared with wildtype ATXN3. HEK-293 cells were transfected with plasmids coding for Flag-tagged ATXN3-Q27 or -Q56 and empty vector as control. At 48 hrs post-transfection cell extracts were analyzed by western blotting. (**d,e**) Exogenous expression of mutant ATXN2, wildtype or mutant TDP-43 recapitulates STAU1 abundances. HEK-293 cells were transfected with plasmids coding for Flag-tagged wildtype or mutant ATXN2, wildtype or mutant TDP-43 or empty vectors as control. 48 hr post-transfection cell extracts were analyzed by western blotting. (**d**) Exogenous mutant ATXN2 expression increases levels of STAU1 compared to control and Flag-ATXN2-Q22. DDX6 levels are not changed. (**e**) Exogenous wildtype or mutant TDP-43 results in increased STAU1 levels compared to control. β-Actin was used as a loading control; blots are from three replicate experiments.

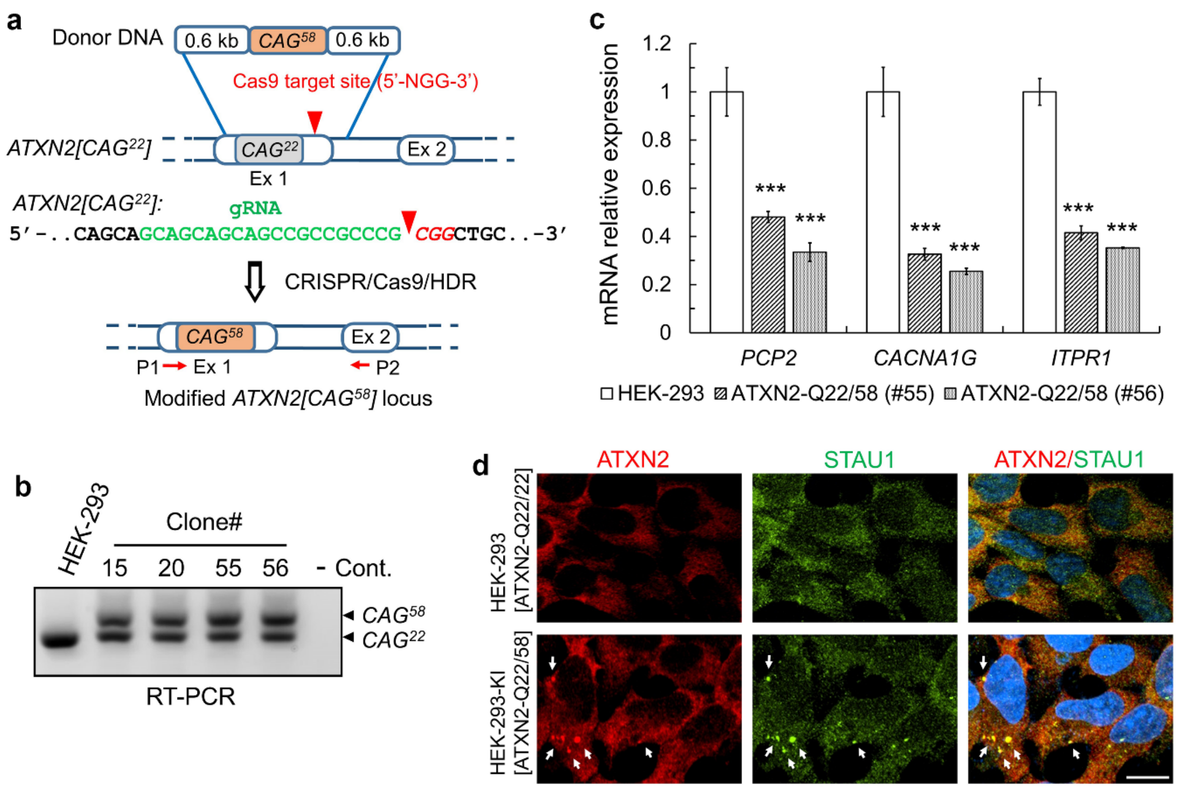

Supplementary Figure 3 | Strategy to knock-in *CAG*^{58} (Q58) repeats into the *ATXN2* locus in HEK-293 cells using the homology-directed-repair (HDR) method of CRISPR/Cas9 editing. (**a**) The single guide RNA (sgRNA) sequence (green), the PAM sequence (NGG) (red) and Cas9 cleavage site (red arrowhead) are shown. Cas9 cleaves the DNA at the target site with sgRNA guidance. The left and right arms of the target donor ATXN2 fragment insert with 58 CAG repeats is inserted into the *ATXN2* locus by HDR. RT-PCR screening for *ATXN2-CAG*$^{22/58}$ [ATXN2-Q22/58] knock-in-positive clones utilized the indicated primers. (**b**) RT-PCR analyses of identified ATXN2-Q22/58 knock-in clones. (**c**,**d**) CRISPR/Cas9 edited ATXN2-Q22/58 KI cells mirror SCA2 phenotypes including STAU1 abundance (Fig. 3c; western blotting) and dysregulation of transcripts: *PCP2, CACNA1G and ITPR1,* that are also reduced in cerebella of SCA2 mice (Fig. 7 and refs. 8,9) determined by qRT-PCR (**c**). Data are mean ± SD, ***$P < 0.001$, Student t-test. (**d**) ATXN2 and STAU1 co-localize in SG-like aggregates. Representative immunohistochemical images from wildtype and ATXN2-Q22/58 KI cells stained with antibodies directed against ATXN2 (red) and STAU1 (green) showing presence of SG-like structures positive for both ATXN2 and STAU1. All images for a respective antibody were taken at the same exposure times. Scale bar, 30 μM.

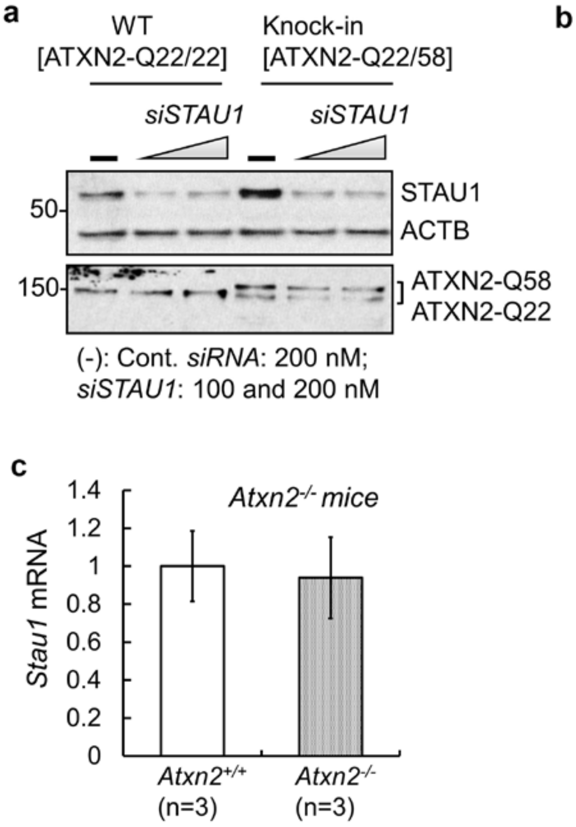

Supplementary Figure 4 | *STAU1* or *ATXN2* reduction in cells and mice without *Atxn2*. (**a**) STAU1 depletion does not affect ATXN2 levels in ATXN2-Q22/58 KI cells. Cells were transfected with *STAU1* siRNA and analyzed by western blotting. (**b**) *ATXN2* silencing lowers STAU1 abundance in ATXN2-Q22/58 KI cells without affecting STAU1 steady-state levels in wildtype cells. Cells were transfected with *ATXN2* RNAi and analyzed by western blotting. β-Actin was used as a loading control and the blots are from three replicate experiments. (**c**) *Stau1* transcript expression is unchanged in cerebella of *Atxn2*$^{-/-}$ mice. qRT-PCR analyses of cerebellar RNAs showing unaltered *Stau1* transcript levels in *Atxn2*$^{-/-}$ mice compared with *Atxn2*$^{+/+}$ mice (8 wks of age; n= 3 animals per group). *Actb* RNA was used as an internal control for qRT-PCR analyses.

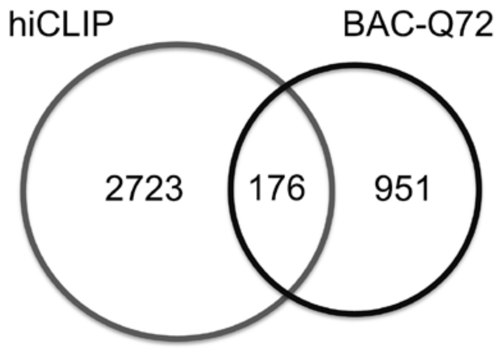

Supplementary Figure 5 | Differentially expressed genes (DEGs) in cerebella of BAC-Q72 mice predicted to bind STAU1. Venn diagram of significant DEGs (AdjP<0.05) in cerebella of 8 wks old BAC-Q72 mice vs wildtype mice [Dansithong *et al*, 2015 (ref. 9)] shared with transcripts interacting with STAU1 determined by hiCLIP [Supplementary Table 2 in Sugimoto *et al*, 2015 (ref. 64)]. A total of 176 transcripts were shared, representing 15.6% of the DEGs in cerebella of BAC-Q72 mice.

Supplementary Table 1 | Differentially expressed genes (DEGs) in cerebella of BAC-Q72 mice [Dansithong *et al*, 2015 (ref. 9)] predicted to bind STAU1, determined by hiCLIP [Sugimoto *et al*, 2015 (ref. 64)].

Gene	Gene	Gene	Gene	Gene	Gene
ABCC4	CERK	FBXO21	MARVELD2	PRPF38B	TLE3
AGFG2	CHAF1A	FGFR2	MAZ	PRPF6	TM7SF3
AGPAT5	CHD7	FNBP1	MCAM	PSAT1	TMCO3
AK4	CHST11	FOXN3	MEGF11	RDX	TMEM109
AMD1	CKB	FOXP4	MFGE8	RGS19	TMEM33
ANAPC5	CLIC1	FREM2	MGAT4B	RGS2	TMEM56
ANLN	CNNM4	GAA	MLLT1	RHOG	TPX2
AP1S2	CNTNAP2	GABBR2	MLLT6	RIMS4	TRAF3
APPL2	CYCS	GAS6	MPRIP	RPS14	TRIM25
ARHGAP5	DAGLA	GJA1	MSX2	SEL1L3	TSPAN14
ASPH	DAP	GNAQ	MUS81	SIPA1L1	UBASH3B
ATP1A1	DHRS11	GNB2L1	MXD4	SIRT2	UNC5B
ATP2B4	DHRS3	GNG12	MYH14	SKI	USP11
ATP5B	DHX9	GNMT	NAB1	SLC16A1	USP28
ATXN7L3B	DNAJA1	GPR85	NFIX	SLC20A1	WAPAL
B2M	DNAJB1	GPT2	NLGN2	SLC25A38	WRB
BBX	DPYSL3	HLCS	NR2F6	SLC38A2	YLPM1
BCAT1	DST	HNRNPA2B1	NRXN2	SLC6A6	YWHAB
BSG	ECE1	HSPH1	P4HA1	SMG5	ZC3H4
BTBD3	EIF2AK2	ICMT	PADI2	SMOC1	ZC3HAV1
BTG1	ELF2	IGFBP5	PAQR7	SNX25	ZRANB1
CAMK2B	EMILIN3	IPO5	PCDH19	SOGA1	
CAMKK1	EMP2	IRF2BP2	PCYOX1L	SPATA2L	
CAMKK2	EN2	IRF2BPL	PEG10	SS18	
CARHSP1	EPHA7	ITGA3	PHF21A	STIP1	
CCT3	ERBB2IP	KLF16	PIK3R3	STK17B	
CCT4	ERC1	LDLRAP1	PLEKHH1	STRN	
CD81	ETS2	LGMN	PMP22	SYT9	
CD83	FADS1	LYPD6	PPFIBP1	TAB3	
CD99L2	FAM107B	MAP3K10	PPM1E	TAB4	
CDC23	FAM83D	MAPRE2	PRKCB	TAB5	

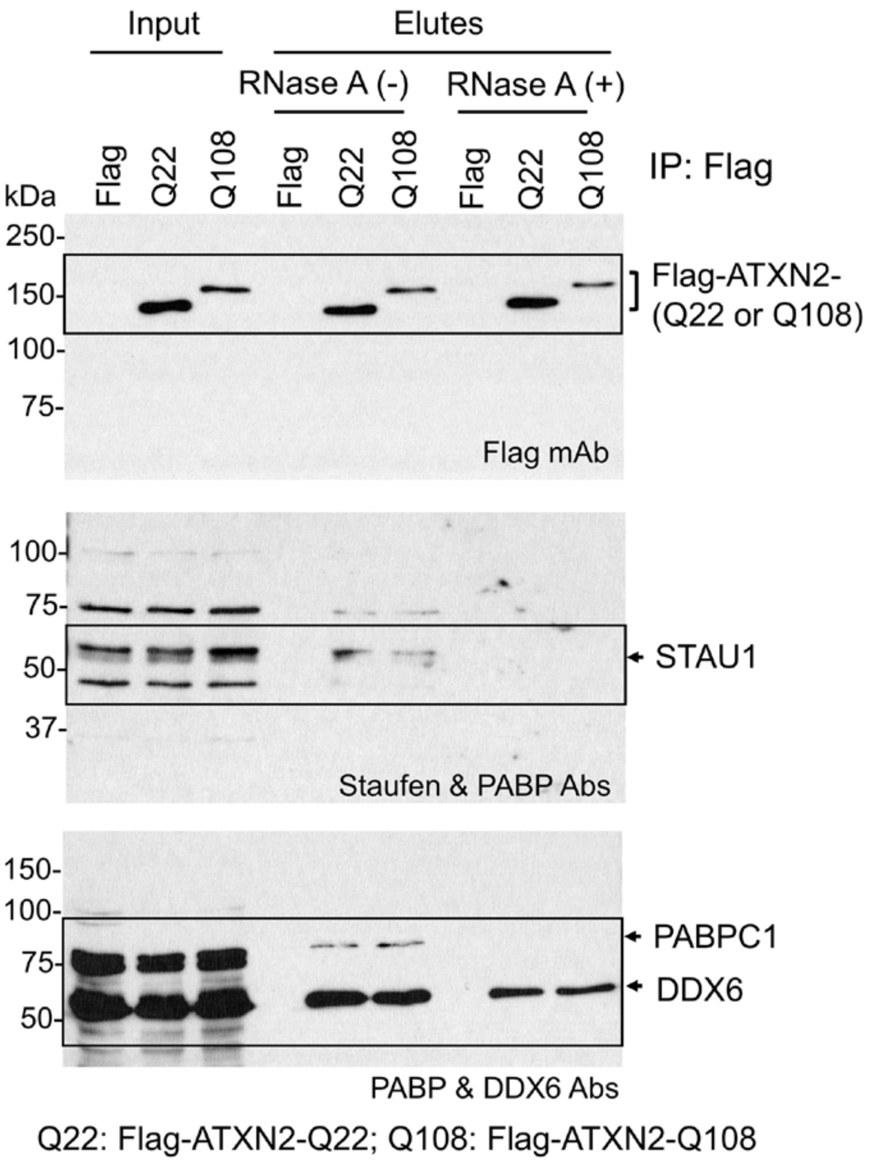

Figure 1g

Supplementary Figure 6 | Detailed western blots for Figure 1g. The relevant figures are indicated in the blots.

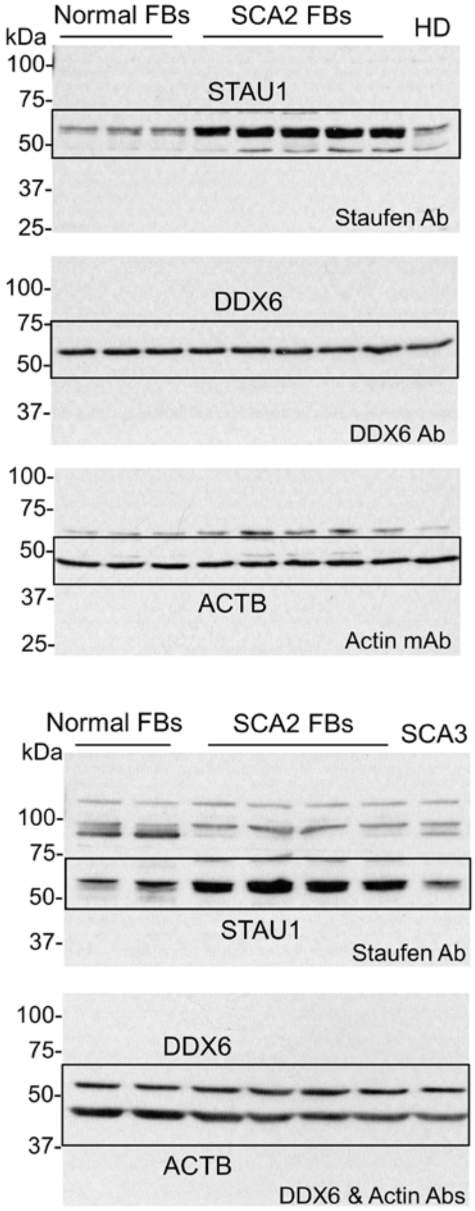

Figure 2a

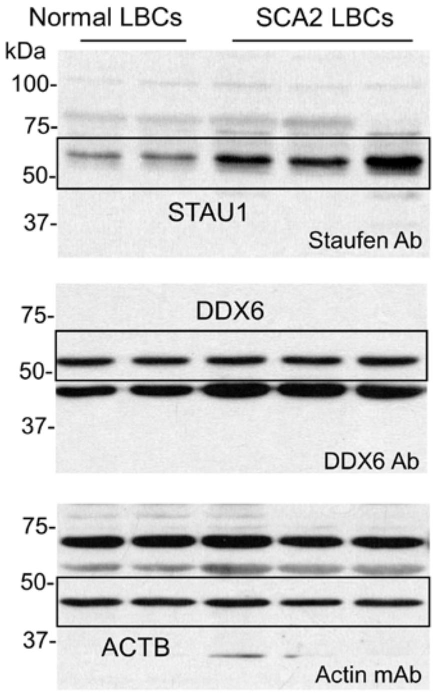

Figure 2b

Supplementary Figure 7 | Detailed western blots for Figures 2a,b. The relevant figures are indicated in the blots.

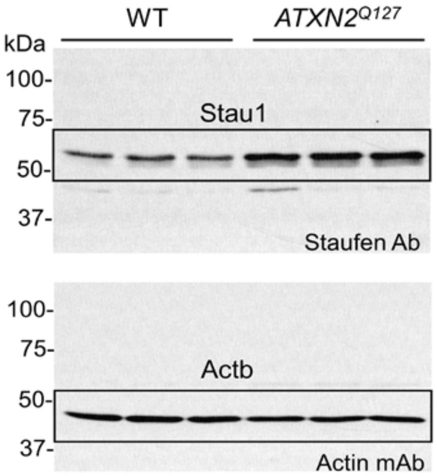

Figure 2c

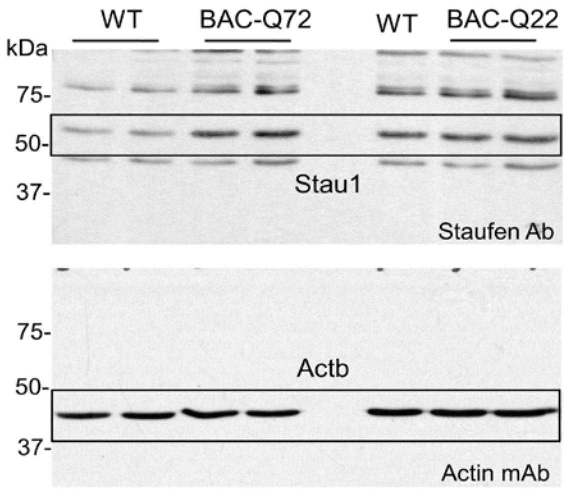

Figure 2d

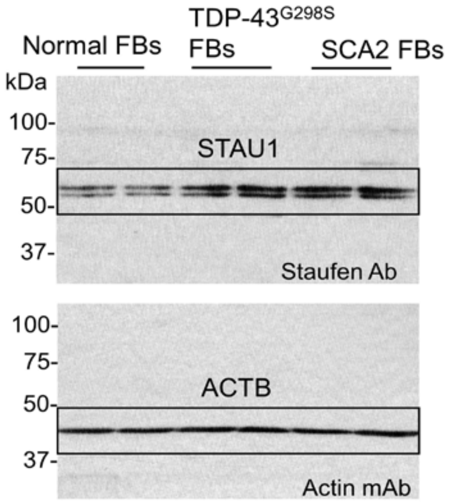

Figure 2e

Supplementary Figure 8 | Detailed western blots for Figures 2c-e. The relevant figures are indicated in the blots.

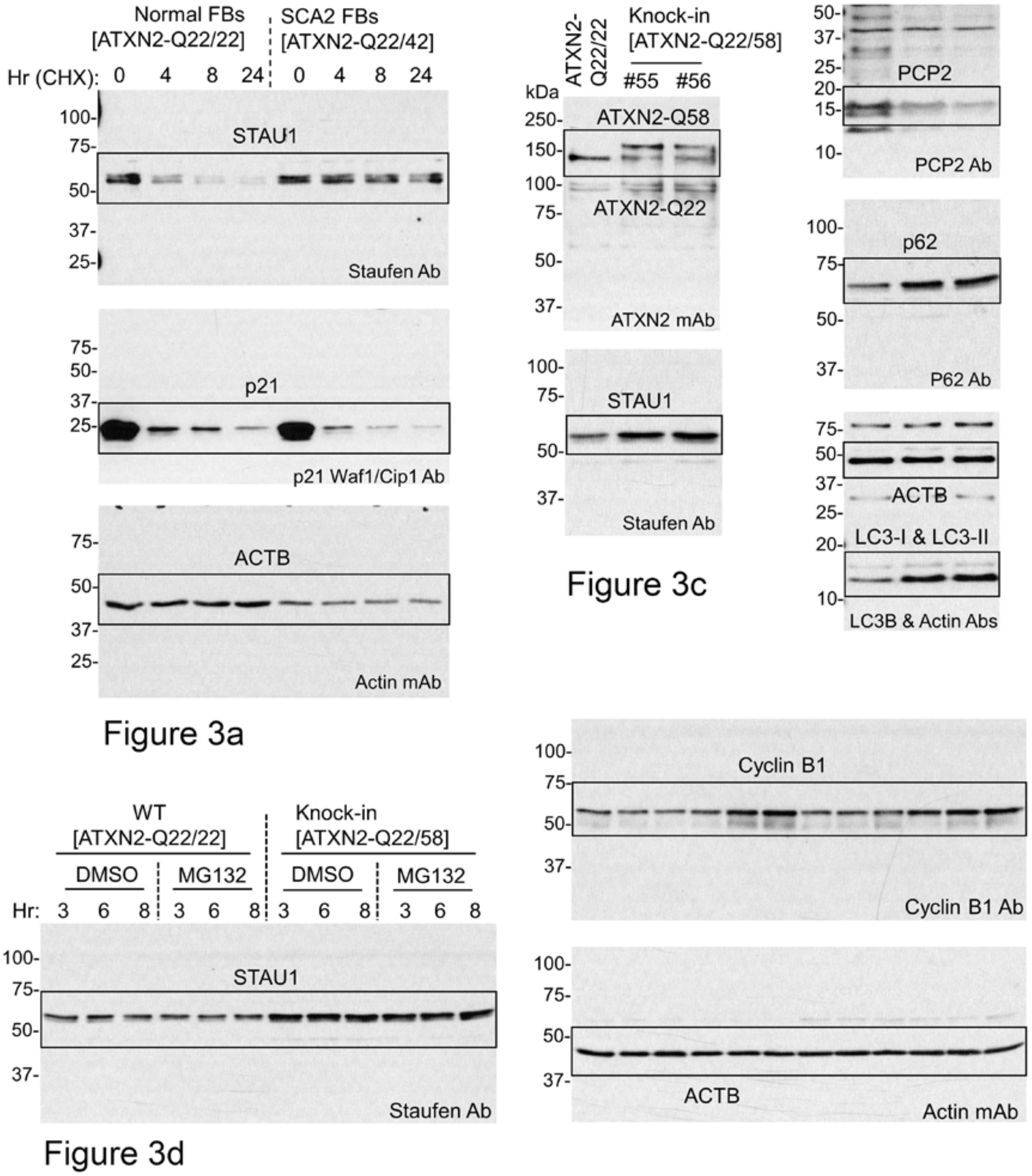

Supplementary Figure 9 | Detailed western blots for Figures 3a,c and d. The relevant figures are indicated in the blots.

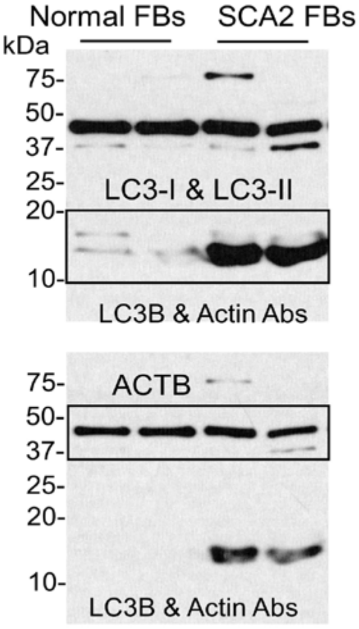

Figure 3f

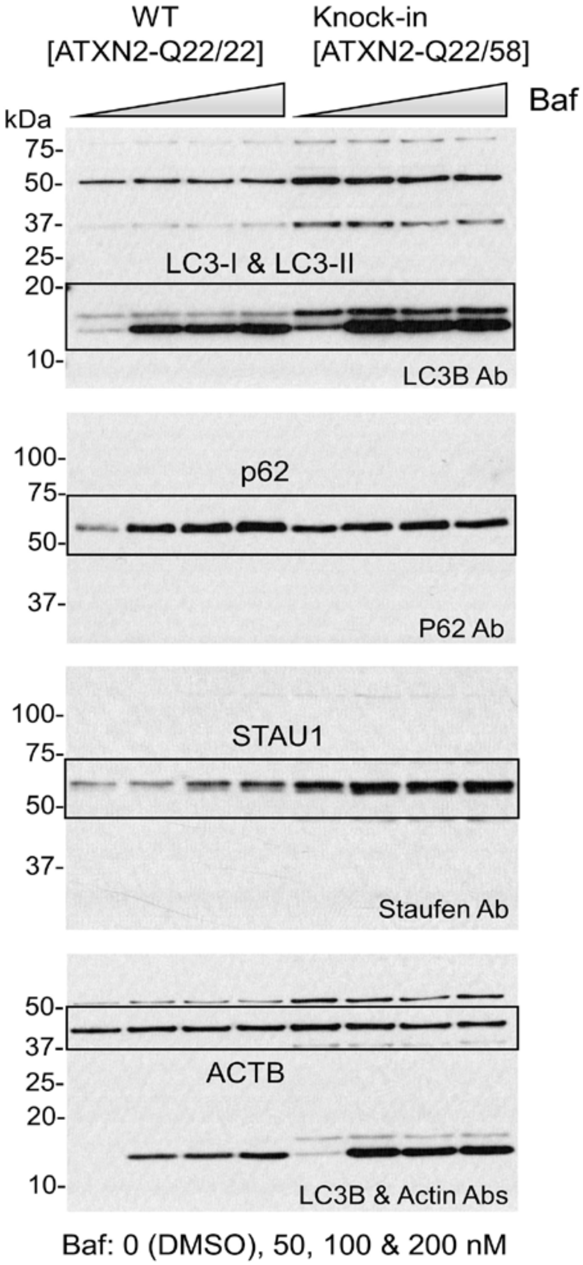

Figure 3g

Supplementary Figure 10 | Detailed western blots for Figures 3f,g. The relevant figures are indicated in the blots.

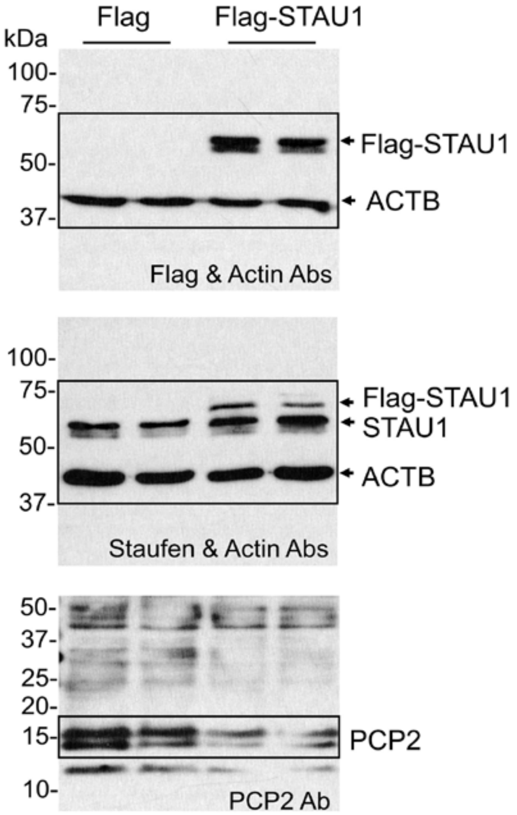

Figure 4c

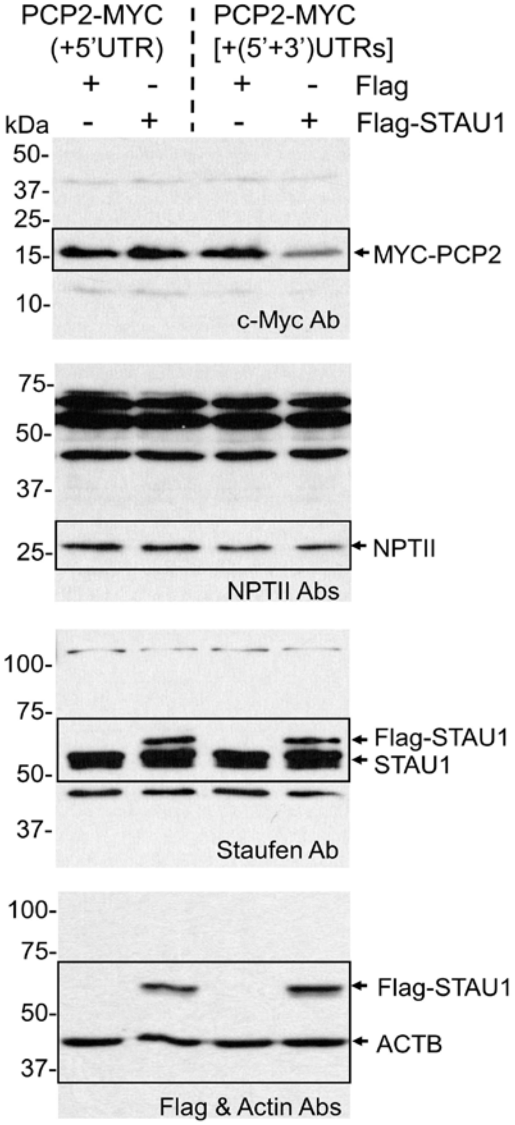

Figure 4g

Supplementary Figure 11 | Detailed western blots for Figures 4c,g. The relevant figures are indicated in the blots.

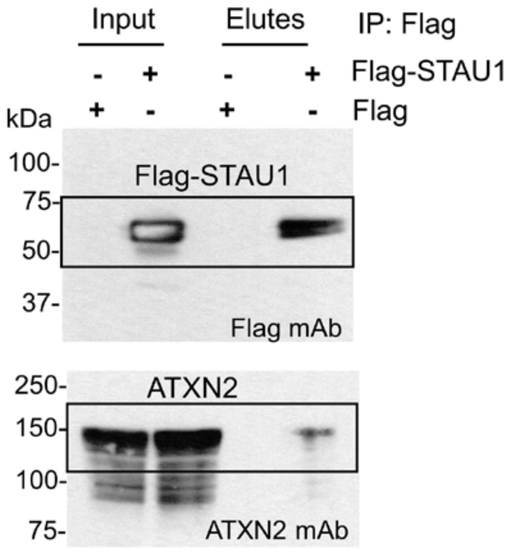

Figure 5a

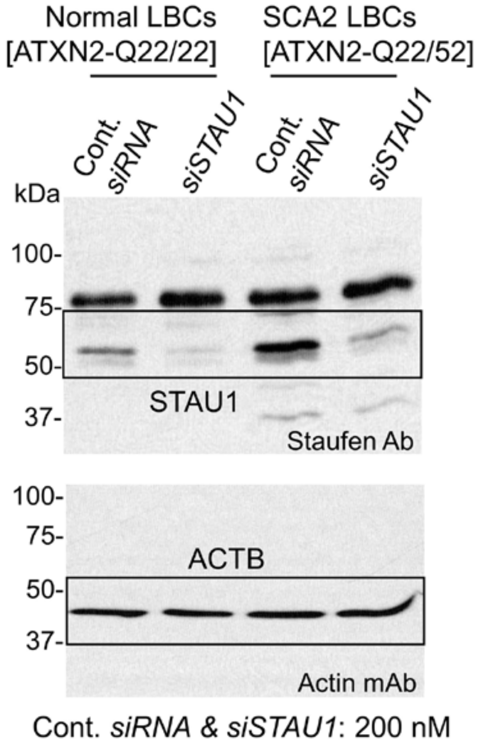

Figure 6a

Supplementary Figure 12 | Detailed western blots for Figures 5a and 6a. The relevant figures are indicated in the blots.

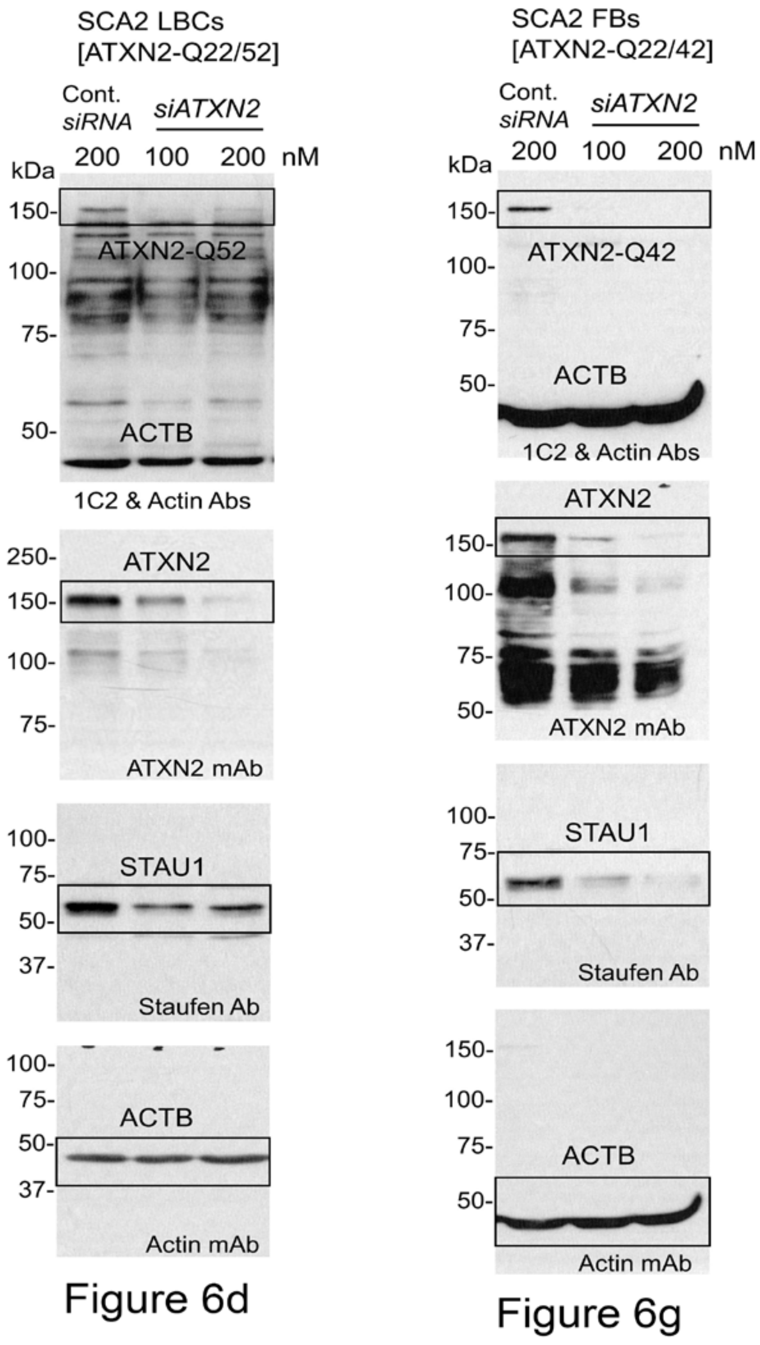

Supplementary Figure 13 | Detailed western blots for Figures 6d,g. The relevant figures are indicated in the blots.

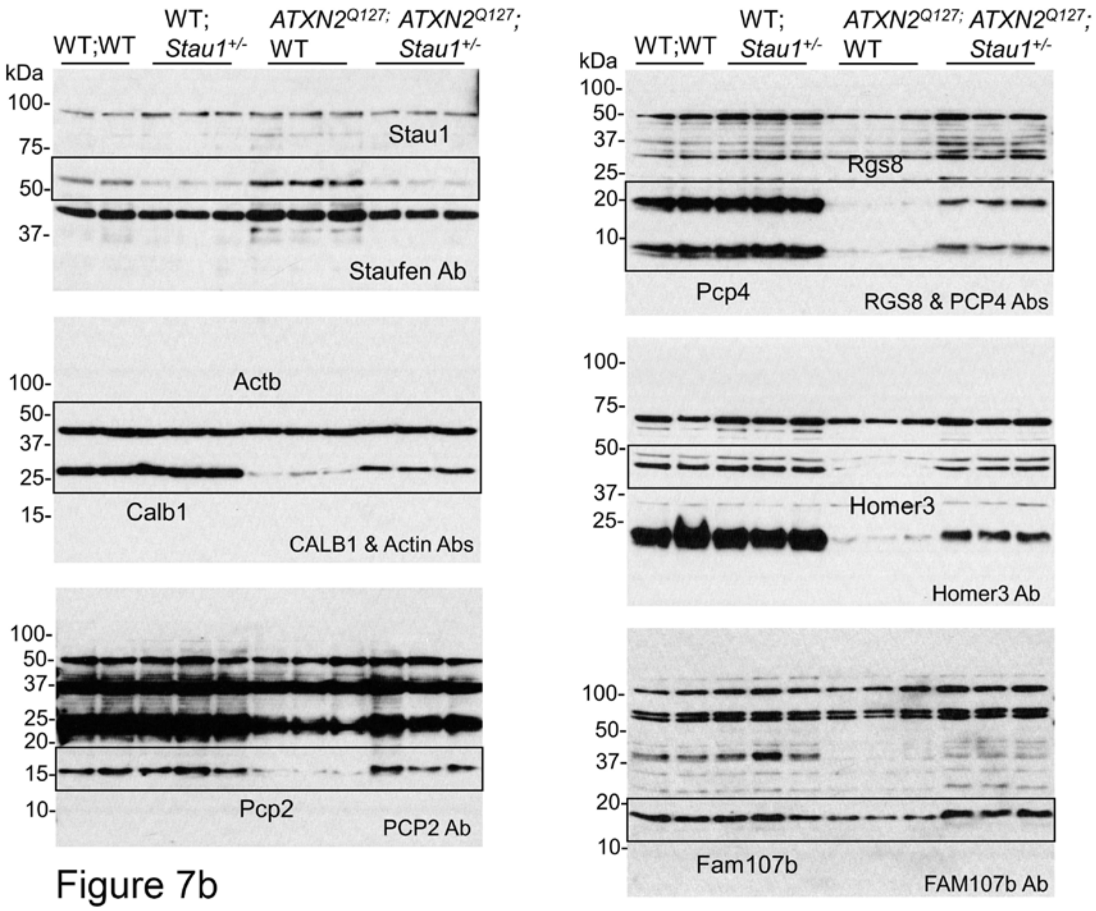

Supplementary Figure 14 | Detailed western blots for Figure 7b. The relevant figures are indicated in the blots.

Supplementary Table 2 | RT-PCR and qPCR primer sequences.

SCA2-A	5'-GGGCCCCTCACCATGTCG-3'	Human	RT-PCR
SCA2-B	5'-CGGGCTTGCGGACATTGG-3'	"	"
ATXN2-Exon1-F	5'CCGCCCGGCGTGCGAGCCGGTGTATGG-3'	"	"
ATXN2-Exon2-R	5'-GTAGACTGAGGCAGTCCTTTGTTACTG-3'	"	"
PCP2-F	5'-GCCAGATCCAGCATCGTTGT-3'	"	"
PCP2-R	5'-CTCTGGCTCTTGGTGGTCTG-3'	"	"
CALB1-F	5'-CAGCAGCTGAAGTCCTGTGA-3'	"	"
CALB1-R	5'-TACAGCTTCCCTCCATCCGA-3'	"	"
GAPDH-F	5'-ACATCGCTCAGACACCATG-3'	"	RT-PCR/qRT-PCR
GAPDH-R	5'-TGTAGTTGAGGTCAATGAAGGG-3'	"	"
ACTB-F	5'-GAAAATCTGGCACCACACCT-3'	"	qRT-PCR
ACTB-R	5'-TAGCACAGCCTGGATAGCAA-3'	"	"
STAU1-F	5'-TCCTTGGTTTCAAAGTCCCG-3'	"	"
STAU1-R	5'-ATTTTCATCCCCAGAGCCAG-3'	"	"
PCP2-F	5'-AAGGACGGAGCACAGAAAC-3'	"	"
PCP2-R	5'-GAGTGAGACCCAGGATGC-3'	"	"
ATXN2-F	5'-AAGATATGGACTCCAGTTATGCAAA-3'	"	"
ATXN2-R	5'-CAAAGCCTCAAGTTCCTCAT-3'	"	"
CACNA1G	5'-CCGACCCACAGATCCCTCTA-3'	"	"
CACNA1G	5'-GCTGTCATTGGGCAGAGAGT-3'	"	"
ITPR1	5'-GCACGTCTTCCTGAGAACCA-3'	"	"
ITPR1	5'-CACTGAGGGCTGAAACTCCA-3'	"	"
Stau1-F	5'-AGTACATGCTCCTTACAGAACG-3'	Mouse	"
Stau1-R	5'-TGATGCCCAACCTTTACCTG-3'	"	"
Actb-F	5'-CGTCGACAACGGCTCCGGCATG-3'	"	"
Actb-R	5'-GGGCCTCGTCACCCACATAGGAG-3'	"	"
